# Perception of medical students about courses based on peer-assisted learning in five Peruvian universities

**DOI:** 10.1186/s13104-020-05237-5

**Published:** 2020-08-20

**Authors:** Anderson N. Soriano-Moreno, Jose E. Delgado-Raygada, C. Ichiro Peralta, Estefania S. Serrano-Díaz, Jaquelin M. Canaza-Apaza, Carlos J. Toro-Huamanchumo

**Affiliations:** 1 Sociedad Científica de Estudiantes de Medicina de la Universidad Peruana Unión, Universidad Peruana Unión, Lima, Peru; 2 Sociedad Científica de Estudiantes de Medicina de la Universidad Peruana de Ciencias Aplicadas, Universidad Peruana de Ciencias Aplicadas, Lima, Peru; 3 Sociedad Científica de Estudiantes de Medicina Villarrealinos, Universidad Nacional Federico Villarreal, Lima, Peru; 4 Sociedad Científica de Estudiantes de Medicina de la Universidad Nacional de Trujillo, Universidad Nacional de Trujillo, Trujillo, Peru; 5 Clínica Avendaño, Lima, Peru; 6 ASME, Association for the Study of Medical Education, Edinburgh, UK

**Keywords:** Peer group, Learning, Medical students, Medical education, Peru

## Abstract

**Objectives:**

Peer-assisted learning (PAL) is a supportive strategy in medical education. In Peru, this method has been implemented by few universities. However, there are no consistent studies evaluating their acceptability by medical students. The objective of this study was to evaluate the perception of medical students about PAL in five Peruvian universities.

**Results:**

A total of 79 medical students were included in the study. The mean age was 20.1 ± 1.9 years, 54% were female, and 87% were in the first 4 years of study. Most of the students were satisfied with classes and peer teachers. Similarly, most of the students agreed with the interest in developing teaching skills. It was also observed that 97% of students approved to implement PAL in medical education programs.

## Introduction

Peer-assisted learning (PAL) is a supportive strategy that consists of students helping their peers to learn while they are involved in the learning process by teaching [1]. Over the years, PAL has become part of medical education programs in different countries and has been identified as an enriching and effective learning method [2–5].

In the United States, 76% of medical schools have implemented this methodology as a complementary activity of regular career courses [6]. In Latin America, PAL has been recently developed as part of educational reforms to improve teaching quality [7].

This innovative method has demonstrated benefits for both peer teachers and learners. Some of the benefits for peer teachers are the development of complex teaching skills, leadership skills, communicative skills, a gain in self-confidence and learning consolidation [1, 3, 4, 8, 9]. The most important advantages for peer learners are the reinforcement of clinical knowledge, the facilitation of the learning process and the enhancement of their academic level [1, 3, 9, 10].

In Peru, the Academic Standing Committee, part of the Sociedad Cientifica Medico Estudiantil Peruana (SOCIMEP), has been promoting the implementation of academic tools with innovative and highly effective methods in many affiliated medical schools. Among these tools, activities using PAL seemed to have achieved high acceptance by medical students. However, there is a lack of objective measurements about the real effectiveness of PAL, as well as its impact on the students’ satisfaction. Therefore, this study aimed to evaluate the perception of medical students about PAL in five Peruvian universities.

## Main text

### Methods

#### Study design

We conducted a survey-based study to evaluate the participants’ perception of reinforcement courses developed with the PAL method in five Peruvian medical schools, from January to March 2018.

#### Sample size and sampling methods

The surveys were applied to the entire target population in a period of 7 days.

#### Eligibility criteria

At the beginning of the year, universities that planned to take courses based on PAL were invited to participate in the study. A total of five universities were included since they developed this activity between January and March.

#### Format of the courses

PAL-based courses covered different topics related to basic medical sciences. All of them followed the recommendations provided by the Association of Medical Education in Europe (AMEE) guidelines for proper planning and implementation of PAL in medical schools [11].

#### Selection of peer teachers

Selection of peer teachers was conducted in each university and prior to course planning. Initially, an announcement was published for the recruitment of students interested in teaching. The students went through an evaluation process, which consisted of making a ten-minute model class. Those who reached subject knowledge, conveyed clearly the information, followed a teaching method and used interactive materials (e.g., slides, videos, or board) were finally selected.

#### Instrument

We used a questionnaire to collect data from our participants. Because there was no validated tool, we designed an instrument based on items from published papers and recommendations given in the literature [12–14]. The questionnaire was reviewed by a professor with expertise in medical education who led the initiation of PAL in one of the universities included in the study. Also, a Cronbach’s alpha was calculated to evaluate the internal consistency of the questionnaire in a sample of twenty medical students, resulting in acceptable reliability for the first and second subsection (0.78 and 0.73 respectively), good reliability for the third and fourth (0.84 and 0.87 respectively) and good reliability for all the questionnaire (0.86, 95% CI 0.75–0.93).

The questionnaire had a three-section structure. Section A compressed sociodemographic characteristics (age, sex and study year). Section B compressed 28-Likert scale questions divided into four subsections (satisfaction with the course, satisfaction with the teacher’s performance, motivation to develop teaching skills and preference to implement PAL in the medical school curriculum). Section C compressed three open questions (What did you like the most about the courses? What could be improved? How was the experience of learning from another student?) that complemented the answers from section B (Additional file 1: Appendix S1).

#### Analysis and presentation of data

Results were introduced into a Microsoft Excel spreadsheet and then exported to Stata 14.0 (StataCorp LLC, TX, USA). Descriptive analysis was used for the sociodemographic characteristics, and mean scores for each Likert-scale item were expressed in tables using means and medians. The answers from open questions were reviewed and categorized into common-word groups.

#### Ethics

The Institutional Review Board of the Universidad Peruana Union approved the study with resolution No. 2018-101. Participation was voluntary and anonymous. Through the participation and completion of the questionnaire, the students authorized their inclusion in the study.

### Results

A total of 79 medical students were included in the study (response rate: ~ 80%). The mean age was 20.1 ± 1.9 years, 54.4% were female, and 87.3% were in the first 4 years of study (Additional file 2: Appendix S2 (Table S1)).

#### Perception of medical students about PAL

In the first subsection of section B, nine statements related to positive facts in the class had an average score higher than 3.99 and medians between 4 (Agree) and 5 (Strongly agree). Negative statements were answered with low scores. Statements “I would have preferred classes to be done by a teacher” and “Only a doctor should teach these subjects” obtained a median of 3 (Neutral) and 2 (Disagree), respectively (Table 1).

**Table 1:** Satisfaction of medical students with peer-assisted learning courses

Question/objective	Mean (SD)^a^	Median^a^	Univ 1.^b^	Univ 2	Univ 3	Univ. 4	Univ 5
The course met the expectations set at the beginning	4.27 (0.59)	Agree	4.29 (0.56)	4.23 (0.44)	4.43 (0.51)	4.57 (0.53)	3.94 (0.75)
The lessons were interesting and enjoyable	4.46 (0.55)	Agree	4.29 (0.64)	4.46 (0.52)	4.62 (0.50)	4.57 (0.53)	4.41 (0.51)
These types of sessions will help me improve my academic performance	4.57 (0.52)	Strongly agree	4.57 (0.60)	4.38 (0.51)	4.76 (0.44)	4.71 (0.49)	4.41 (0.51)
I was able to directly apply what I learned	4.35 (0.58)	Agree	4.38 (0.59)	4.31 (0.63)	4.48 (0.51)	4.57 (0.53)	4.12 (0.60)
Theory and practice were well combined	3.99 (0.74)	Agree	3.95 (0.50)	4.15 (0.69)	4.48 (0.51)	3.57 (1.27)	3.47 (0.62)
I would have preferred the classes to be done by a regular teacher	3.01 (0.85)	Neutral	3.19 (0.87)	2.62 (0.87)	3.14 (0.85)	2.86 (0.90)	3.00 (0.79)
Group size was optimal	4.08 (0.78)	Agree	3.86 (0.91)	3.92 (0.76)	4.29 (0.72)	4.00 (0.82)	4.24 (0.66)
Only a doctor could teach this	2.63 (1.13)	Disagree	2.95 (1.20)	2.31 (1.11)	2.71 (1.10)	2.43 (1.27)	2.47 (1.07)
Time spent in the process was appropriate	4.09 (0.72)	Agree	3.81 (0.93)	4.31 (0.63)	4.38 (0.50)	4.00 (0.82)	3.94 (0.56)
I would recommend the sessions using the PAL methodology	4.39 (0.56)	Agree	4.33 (0.58)	4.31 (0.75)	4.43 (0.51)	4.86 (0.38)	4.29 (0.47)
I am willing to attend another similar session	4.59 (0.52)	Strongly agree	4.57 (0.60)	4.62 (0.51)	4.57 (0.51)	4.86 (0.38)	4.53 (0.51)

*SD* Standard deviation,* Univ* University,* PAL* peer assisted learning

^a^1 = Strongly disagree, 2 = Disagree, 3 = Neutral, 4 = Agree, 5 = Strongly agree

^b^Mean obtained for each of the five universities

Regarding the satisfaction with the peer teachers, positive results were observed. In all the positive statements, scores greater than 4 (Agree) were obtained. Only in the negative statement “There were questions that the peer-teacher could not answer” the median obtained was 3 (Neutral) (Table 2).

**Table 2:** Satisfaction of medical students regarding the peer teacher

Question/objective	Mean (SD)^a^	Median^a^	Univ 1.^b^	Univ 2	Univ 3	Univ 4	Univ 5
Peer teacher demonstrated mastery of the subject	4.41 (0.57)	Agree	4.29 (0.64)	4.54 (0.66)	4.48 (0.51)	4.57 (0.53)	4.29 (0.47)
He/She showed similar skills or better than usual teachers	4.16 (0.72)	Agree	4.14 (0.85)	4.38 (0.65)	4.19 (0.75)	4.14 (0.69)	4.00 (0.61)
Clarity of the explanations	4.32 (0.52)	Agree	4.33 (0.58)	4.38 (0.51)	4.38 (0.50)	4.43 (0.53)	4.12 (0.49)
Comfort in asking questions	4.10 (0.65)	Agree	3.95 (0.67)	4.31 (0.63)	4.24 (0.62)	4.43 (0.53)	3.82 (0.64)
Interaction between students and teacher was good	4.29 (0.58)	Agree	4.33 (0.58)	4.23 (0.73)	4.43 (0.60)	4.43 (0.53)	4.06 (0.43)
The information provided was based on updated articles	4.14 (0.71)	Agree	3.90 (0.77)	4.54 (0.52)	4.29 (0.78)	4.29 (0.49)	3.88 (0.60)
The class was appropriately organized	4.27 (0.61)	Agree	4.10 (0.77)	4.38 (0.65)	4.43 (0.51)	4.57 (0.53)	4.06 (0.43)
He / She was able to teach complex subjects in a simple way	4.25 (0.59)	Agree	4.19 (0.68)	4.31 (0.75)	4.33 (0.48)	4.14 (0.69)	4.24 (0.44)
He / She used interactive resources that helped to strengthen my learning	4.29 (0.64)	Agree	4.33 (0.80)	4.31 (0.63)	4.29 (0.72)	4.29 (0.49)	4.24 (0.44)
Questions asked were appropriately answered	4.19 (0.51)	Agree	4.19 (0.51)	4.31 (0.63)	4.30 (0.47)	4.00 (0.58)	4.06 (0.43)
There were many unanswered questions	2.82 (1.07)	Neutral	2.67 (1.15)	2.77 (1.17)	3.15 (1.18)	2.57 (0.79)	2.76 (0.83)

*SD* Standard deviation,* Univ* University

^a^1 = Strongly disagree, 2 = Disagree, 3 = Neutral, 4 = Agree, 5 = Strongly agree

^b^Mean obtained for each of the five universities

Regarding the motivation of the participants to be peer teachers in the future and to develop teaching skills, 78.5% (62/79), 93.7% (74/79) and 89.9% (71/79) scored higher than 4 (Agree) the statements “The course encouraged me to practice teaching in the future”, “The practice of undergraduate teaching would be very beneficial for my professional development” and “I would like to attend to a session to learn teaching skills” (Table 3).

**Table 3:** Motivation of medical students to develop skills in teaching and interest in implementing the PAL method

Question/Objective	Mean (SD)^a^	Median^a^	Univ 1. ^b^	Univ 2	Univ 3	Univ. 4	Univ 5
Motivation of the assistants to develop skills in teaching							
The session has encouraged me to practice student teaching in the future	4.10 (0.73)	Agree	4.24 (0.83)	4.00 (0.71)	4.14 (0.79)	4.29 (0.76)	3.88 (0.49)
I think that doing peer teaching would be very beneficial for my professional development	4.34 (0.60)	Agree	4.38 (0.59)	4.38 (0.51)	4.43 (0.68)	4.29 (0.76)	4.18 (0.53)
I would like to attend a session where teaching skills are taught	4.34 (0.66)	Agree	4.48 (0.60)	4.46 (0.52)	4.43 (0.75)	4.57 (0.53)	3.88 (0.60)
Interest in implementing the PAL method to the university curriculum							
I believe that students with teaching skills can offer a class of similar or better quality than regular teachers	4.35 (0.64)	Agree	4.48 (0.51)	4.31 (0.75)	4.43 (0.75)	4.14 (0.69)	4.24 (0.56)
It would be useful to implement courses using this methodology in the usual curriculum to reinforce the most complicated courses	4.47 (0.53)	Agree	4.48 (0.51)	4.54 (0.52)	4.57 (0.51)	4.29 (0.76)	4.35 (0.49)
I would agree that PAL classes are held on vacation	4.44 (0.55)	Agree	4.33 (0.66)	4.38 (0.51)	4.57 (0.51)	4.57 (0.53)	4.41 (0.51)

*SD* Standard deviation,* Univ* University,* PAL* peer assisted learning

^a^1 = Strongly disagree, 2 = Disagree, 3 = Neutral, 4 = Agree, 5 = Strongly agree

^b^Mean obtained for each of the five universities

In the subsection of participants’ interest to implement PAL in medical schools, we found that 98.7% (78/79) scored higher than 4 (Agree) the statement “I would like to see classes implemented through PAL in summer”. Also, 97.4% (77/79) rated the statement “It would be useful to implement PAL-based courses in the university curriculum” with scores higher than 4 (Table 3).

#### Answers to open questions

The most frequently observed response regarding what the students liked the most about the course was the “teaching and didactic of the peer-teacher” (32.9%). The method and structure of the course were also considered of high value by 14 participants (17.7%). When participants were asked about what could be improved about PAL-based courses, 18 (22.8%) students answered “nothing”, 11 (14.1%) said that longer courses are needed, and 11 (14.1%) indicated that other educational resources should be used. Finally, in the question that assessed what students think about learning from another student, 35 answers (44.3%) were observed with the words “Very good, good, and incredible” and another 15 (18.9%) related with the peer teacher’s empathy and the confidence that they provided (Additional file 2: Appendix S2 (Table S2)).

### Discussion

We evaluated the perception of medical students about courses using the PAL strategy in five medical schools in Peru. We observed that students had positive perceptions about the program, peer teacher, motivation to be a peer teacher, and interest to implement this approach into the regular curriculum. These results are a good approach to the benefits of developing PAL, being consistent with previous reports [1–6, 9, 10, 12, 14]. However, systematic reviews have not shown enough evidence to demonstrate its efficacy in some aspects [3, 15].

PAL-based courses promote the social congruence between the peer teacher and the learner in a comfortable interaction environment [1, 3, 4]. This could explain why students often feel satisfied with this strategy, as we presented in our study. Another possible explanation might be that the peer teacher and the students share a similar academic background. It was observed that students valued the learning from peer teachers because they understand the students’ struggles in medical school and the shared experiences are valuable assets [1].

We also found that participants had a positive attitude towards becoming peer teachers. Previous studies have reported the advantages of achieving this, which include a better understanding of the topic being taught and leadership development [1, 16]. Therefore, it should be considered as an opportunity for self-improvement. Our study showed that after the PAL-based sessions, the participants were motivated to learn teaching skills and considered that this would be a benefit for their professional development. Similar results have been reported in previous studies, and the awareness of the benefits of PAL among medical students probably encourages their training to become peer-teachers [1, 17, 18].

Participants also agreed with the implementation of PAL in their regular curriculum. This might be a reflection of the need to use novel medical teaching methods in highly difficult courses to improve academic performance [19]. These could include learning support interventions, implementation of small working groups, training in coping strategies and learning styles, and a constant evaluation of acquired knowledge. In addition, peer teachers could be more prepared than faculty professors to teach review classes. They have a better perception of academic pitfalls and, since both the peer teacher and learners had probably experienced the same academic challenges, a more empathic learning environment could be achieved.

### Conclusion

In our study, medical students had a positive perception of PAL. The quality of classes and the peer teachers' performance were relevant for the results. Moreover, we present the first study about PAL as a strategy to overcome crucial deficiencies in medical education in Peru. A study with a better sample size would be necessary to verify our results. However, we suggest that PAL should be implemented in Peruvian medical schools as part of their curriculum.

## Limitations

First, we did not find an adequate instrument that assessed all our variables of interest; hence, we designed and developed a questionnaire instead of using a previously validated instrument. However, we tested the reliability of the survey questions, obtaining good values for Cronbach’s alphas. Second, the small sample size was also a limitation.

## Availability of data and materials

Although all the data generated or analyzed during this study are included in this manuscript, the datasets are available from the corresponding author upon reasonable request.

## Supplementary information

**Additional file 1: Appendix S1.** Questionnaire developed for this study.

**Additional file 2: Appendix S2.** Extra results tables. Contains the **Table S1.** General characteristics of the students surveyed and the **Table S2.** Summary of the most common free-text responses.

## Data Availability

Although all the data generated or analyzed during this study are included in this manuscript, the datasets are available from the corresponding author upon reasonable request.

## Abbreviations

PAL: Peer-assisted learning
SOCIMEP: Sociedad Científica Médico Estudiantil Peruana
AMEE: Association of Medical Education in Europe
SD: Standard deviation
