# Supplementary material for: Perception of medical students about courses based on peer-assisted learning in five Peruvian universities
Source: BMC Res Notes. 2020 Aug 20;13:391. doi: 10.1186/s13104-020-05237-5 (PMC7441666; doi:10.1186/s13104-020-05237-5)
Supplement: Supplementary file 1 — Additional file 1: Appendix S1. Questionnaire developed for this study. [file 13104_2020_5237_MOESM1_ESM.docx]

# Assessment of the perception of medical students about courses based on peer-assisted learning

Dear student, this survey has the purpose of assessing the perceptions of medical students regarding the peer-assisted learning methodology-based courses. From the results obtained, the quality of these courses can be improved and a research study will be carried out. Participation is completely voluntary and will take approximately 10 minutes of your time. If you agree to participate, please answer the next questions by putting an "X" in the boxes according to your opinion. The results will be completely anonymous. Thank you very much for your participation!

1. **General Information**

Age (years): _____

Gender:

|  | Male |
| --- | --- |
|  |  |
|  | Female |

Study year:

|  | 1^st^ year |
| --- | --- |
|  |  |
|  | 2^nd^ year |
|  |  |
|  | 3^rd^ year |

|  | 4^th^ year |
| --- | --- |
|  |  |
|  | 5^th^ year |
|  |  |
|  | 6^th^ year |

**B.1 Regarding the courses based on Peer-Assisted Learning methodology**

|  | Strongly disagree | Disagree | Neutral | Agree | Strongly agree |
| --- | --- | --- | --- | --- | --- |
| 1. The course met the expectations set at the beginning. |  |  |  |  |  |
| 1. The lessons were interesting and enjoyable. |  |  |  |  |  |
| 1. These types of sessions will help me improve my academic performance. |  |  |  |  |  |
| 1. I was able to directly apply what I learned. |  |  |  |  |  |
| 1. Theory and practice were well combined. |  |  |  |  |  |
| 1. I would have preferred the classes to be done by a regular teacher |  |  |  |  |  |
| 1. Group size was optimal. |  |  |  |  |  |
| 1. Only a doctor could teach this. |  |  |  |  |  |
| 1. Time spent in the process was appropriate. |  |  |  |  |  |
| 1. I would recommend the sessions using the PAL methodology |  |  |  |  |  |
| 1. I am willing to attend another similar session. |  |  |  |  |  |

**B.2 Regarding the peer teacher**

|  | Strongly disagree | Disagree | Neutral | Agree | Strongly agree |
| --- | --- | --- | --- | --- | --- |
| 1. Peer teacher demonstrated mastery of the subject. |  |  |  |  |  |
| 1. He / She showed similar skills or better than usual teachers. |  |  |  |  |  |
| 1. Clarity of the explanations. |  |  |  |  |  |
| 1. Comfort in asking questions. |  |  |  |  |  |
| 1. Interaction between students and teacher was good. |  |  |  |  |  |
| 1. The information provided was based on updated articles. |  |  |  |  |  |
| 1. The class was appropriately organized. |  |  |  |  |  |
| 1. He / She was able to teach complex subjects in a simple way. |  |  |  |  |  |
| 1. He / She used interactive resources that helped to strengthen my learning. |  |  |  |  |  |
| 1. Questions asked were appropriately answered. |  |  |  |  |  |
| 1. There were many unanswered questions. |  |  |  |  |  |

**B.3 Regarding interest to develop teaching skills being a medical student**

|  | Strongly disagree | Disagree | Neutral | Agree | Strongly agree |
| --- | --- | --- | --- | --- | --- |
| 1. The session has encouraged me to practice student teaching in the future. |  |  |  |  |  |
| 1. I think that doing peer teaching would be very beneficial for my professional development. |  |  |  |  |  |
| 1. I would like to attend a session where teaching skills are taught. |  |  |  |  |  |

**B.4 Regarding the implementation of Peer-Assisted Learning methodology in the university curriculum**

|  | Strongly disagree | Disagree | Neutral | Agree | Strongly agree |
| --- | --- | --- | --- | --- | --- |
| 1. I believe that students with teaching skills can offer a class of similar or better quality than regular teachers. |  |  |  |  |  |
| 1. It would be useful to implement courses using this methodology in the usual curriculum to reinforce the most complicated courses. |  |  |  |  |  |
| 1. I would agree that PAL classes are held on vacation. |  |  |  |  |  |

1. **Open-Ended Questions**
2. What did you like the most about the courses?
3. What could be improved?
4. How was the experience of learning from another student?
