# Supplementary material for: Perception of medical students about courses based on peer-assisted learning in five Peruvian universities
Source: BMC Res Notes. 2020 Aug 20;13:391. doi: 10.1186/s13104-020-05237-5 (PMC7441666; doi:10.1186/s13104-020-05237-5)
Supplement: Supplementary file 2 — Additional file 2: Appendix S2. Extra results tables. Contains the Table S1. General characteristics of the students surveyed and the Table S2. Summary of the most common free-text responses. [file 13104_2020_5237_MOESM2_ESM.docx]

# Appendix S2: Extra results tables

| **Table S1. General characteristics of the surveyed students (N = 79)** | |
| --- | --- |
| **Characteristics** | **Total N (%)** |
| Female | 43 (54.4) |
| Age (Mean ± SD)^a^ | 20.1 ±1.9 |
| Scientific society |  |
| University 1 | 21 (26.5) |
| University 2 | 13 (16.4) |
| University 3 | 21 (26.5) |
| University 4 | 7 (8.8) |
| University 5 | 17 (21.5) |
| Year of study |  |
| 1st | 3 (3.8) |
| 2nd | 28 (35.4) |
| 3rd | 15 (19.0) |
| 4th | 23 (29.1) |
| 5th | 4 (5.1) |
| 6th | 6 (7.6) |
| *^a^ SD* = Standard Deviation | |

| **Table S2.** **Summary of the most common free-text responses from 79 students categorized by the question** | |
| --- | --- |
| **Question** | **N (%)** |
| **What did you like the most about the courses?** | **N=61** |
| Tutors teaching skills / Good explanations | 26 (32.9) |
| Well-structured and didactic sessions | 14 (17.7) |
| Use of novel teaching resources | 8 (10.1) |
| Made it easy to ask questions, reliable interaction | 7 (8.9) |
| Reinforcement medical topics, learn new topics. | 6 (7.6) |
| **What could be improved?** | **N=47** |
| Nothing | 18 (22.8) |
| Increase the number and length of activities | 11 (14.1) |
| Use and provision of other additional educational resources | 11 (14.1) |
| More clinical relevance | 7 (8.9) |
| **How was the experience of learning from another student?** | **N=65** |
| Very good, incredible, satisfactory | 35 (44.3) |
| Motivating, inspiring, encouraging to teach | 15 (18.9) |
| Less stressful / more comfortable | 11 (14.1) |
| New experience, good experience, interesting experience. | 8 (10.1) |
| Note: Some students may have commented on >1 parameter |  |
